# Antitumor Activity and Treatment-Related Toxicity Associated With Nivolumab Plus Ipilimumab in Advanced Malignancies: A Systematic Review and Meta-Analysis

**DOI:** 10.3389/fphar.2019.01300

**Published:** 2019-11-04

**Authors:** Hang Xu, Ping Tan, Jianzhong Ai, Shiyu Zhang, Xiaonan Zheng, Xinyang Liao, Lu Yang, Qiang Wei

**Affiliations:** ^1^Department of Urology, Institute of Urology, West China Hospital, Sichuan University, Chengdu, China; ^2^West China Medical School, Sichuan University, Chengdu, China

**Keywords:** nivolumab, ipilimumab, combination, dosage, objective response rate, adverse events

## Abstract

Combining immune checkpoint inhibitors has shown its efficacy compared to monotherapy in advanced malignancies. We conducted this meta-analysis to provide latest evidence on the objective response rate (ORR) and incidence of treatment-related high-grade adverse events (AEs) during nivolumab and ipilimumab combination treatment and further explore from different drug dose level. PubMed and the 2019 American Society of Clinical Oncology (ASCO) annual meeting abstracts were searched for qualified clinical trials up to June 2019. Of the 23 clinical trials (13 from publications and 11 from ASCO abstracts) included, 2,114 and 2,674 patients were eligible for efficacy and safety analysis, respectively. Pooled analysis suggested that the overall ORR was achieved in 34.5% [95% confidence interval (CI), 29.1–40.4%] of patients. There was no significant difference between nivolumab 3 mg/kg plus ipilimumab 1 mg/kg every 3 weeks (N3I1-Q3W) and nivolumab 1 mg/kg plus ipilimumab 3 mg/kg every 3 weeks (N1I3-Q3W) arms in ORR [30.8% vs 41%; odds ratio (OR), 0.72; 95% CI, 0.39–1.30; *P* = 0.275]. Grade 3–4 AEs related to combination therapy occurred in 39.9% (95% CI, 33.5–46.7%) of patients; the most commonly reported grade 3–4 treatment-related AEs were diarrhea (5.28%), colitis (3.96%) and increased alanine aminotransferase (3.51%). Incidence of grade 3–4 AEs were significant lower in N3I1-Q3W arm than in N1I3-Q3W arm (31.3% vs 55.9%; OR 0.52; 95% CI, 0.32–0.87; *P* = 0.012). Treatment-related death was rare and occurred in 2.0% (95% CI, 1.5–2.7%) of patients. Our comprehensive study provides more precise data on the incidence of treatment-related high-grade AEs and ORR among patients receiving nivolumab and ipilimumab combination regimens. Patients on the N3I1-Q3W arm had comparable ORR and significantly occurred less grade 3–4 AEs than patients on the N1I3-Q3W arm. Our finding is of great importance in assisting clinical trial design and clinical medication choice.

## Introduction

Therapeutic strategies for advanced cancers have dramatically evolved over the past decade. As the traditional chemotherapy gradually couldn’t achieve satisfied clinical outcomes in some clinical settings, immune checkpoint inhibitors (ICIs), which specifically target cytotoxic T lymphocyte antigen-4 (CTLA-4) and programmed death-1/ligand-1 (PD-1/PD-L1), have largely altered the treatment predicament in various advanced cancer types (Martins et al., 2019). Compared with monotherapies, combined use of anti-CTLA-4 and anti-PD-1/PD-L1 appears to exert durable response and longer survival benefit in a large proportion of advanced cancer patients (Hodi et al., 2016; Wolchok et al., 2017; Hellmann et al., 2018).

Among all the ICIs, ipilimumab and nivolumab are the most widely used ICI drugs till now, and these two drugs are the earliest and the most frequently used as combination regimens in clinical settings. Ipilimumab is a fully human IgG1 CTLA-4 ICI antibody which block the CTLA-4–B7 interaction and nivolumab is a fully human IgG4 PD-1 ICI antibody which can block the PD-1-PD-L1 interaction between T cells and tumor cells. Both of these two drugs can enhance the T-cell function through different ways in depleting tumor cells and thus might induce clinical response in cancer patients (O’Day et al., 2007; Buchbinder and Desai, 2016).

Accumulating clinical trials has been initiated to evaluate the clinical outcomes of nivolumab plus ipilimumab across various tumor types such as melanoma (Tawbi et al., 2018), lung cancer (Hellmann et al., 2018), renal cell carcinoma (Motzer et al., 2018) and colorectal cancer (Overman et al., 2018). Nevertheless, merely focusing on the response rate and survival benefit brought by the combination use seems insufficient, the treatment-related adverse events (AEs) or immune-related AEs also occur during the ICI treatment. Some AEs were slight and unrecognizable, while other AEs such as grade 3 or more AEs were severe and might lead to treatment discontinuation, hospitalization, and even death (Martins et al., 2019). The frequency and spectrum of high-grade AEs during nivolumab plus ipilimumab combination treatment, however, have not been well investigated. A recent meta-analysis showed that the immunotherapy combination could produce more clinical benefits while with increased high-grade AEs (Wei et al., 2019). Subsequent question was raised that how we clinicians can formulate an optimal combination regimen in reducing the incidence of treatment-related high-grade AEs while not compromising its efficacy at the same time.

Herein, by reviewing the latest evidence in cancer immunotherapy progress, we conducted this meta-analysis trying to exhibit the frequency and spectrum of high-grade/fatal AEs and the objective response rate (ORR) related to nivolumab and ipilimumab combination therapy. We also sought to further explore the outcomes from different drug dose level.

## Materials and Methods

### Search Strategy

We systematically searched the PubMed database to identify the clinical trials that investigated the combined nivolumab and ipilimumab use in cancer patients and report the related results without language restrictions. Besides, the 2019 American Society of Clinical Oncology (ASCO) annual meeting abstracts were also retrieved as potential sources. For PubMed search, the following keywords were used: “Ipilimumab,” “Yervoy,” “MDX-010,” “BMS-734016,” “nivolumab,” “Opdivo,” “BMS-936558,” “MDX1106.” PubMed search was up to June 1, 2019. We only searched the nivolumab and ipilimumab because they are the most frequently used combined ICIs in clinical trials.

### Study Selection

**Figure 1:**
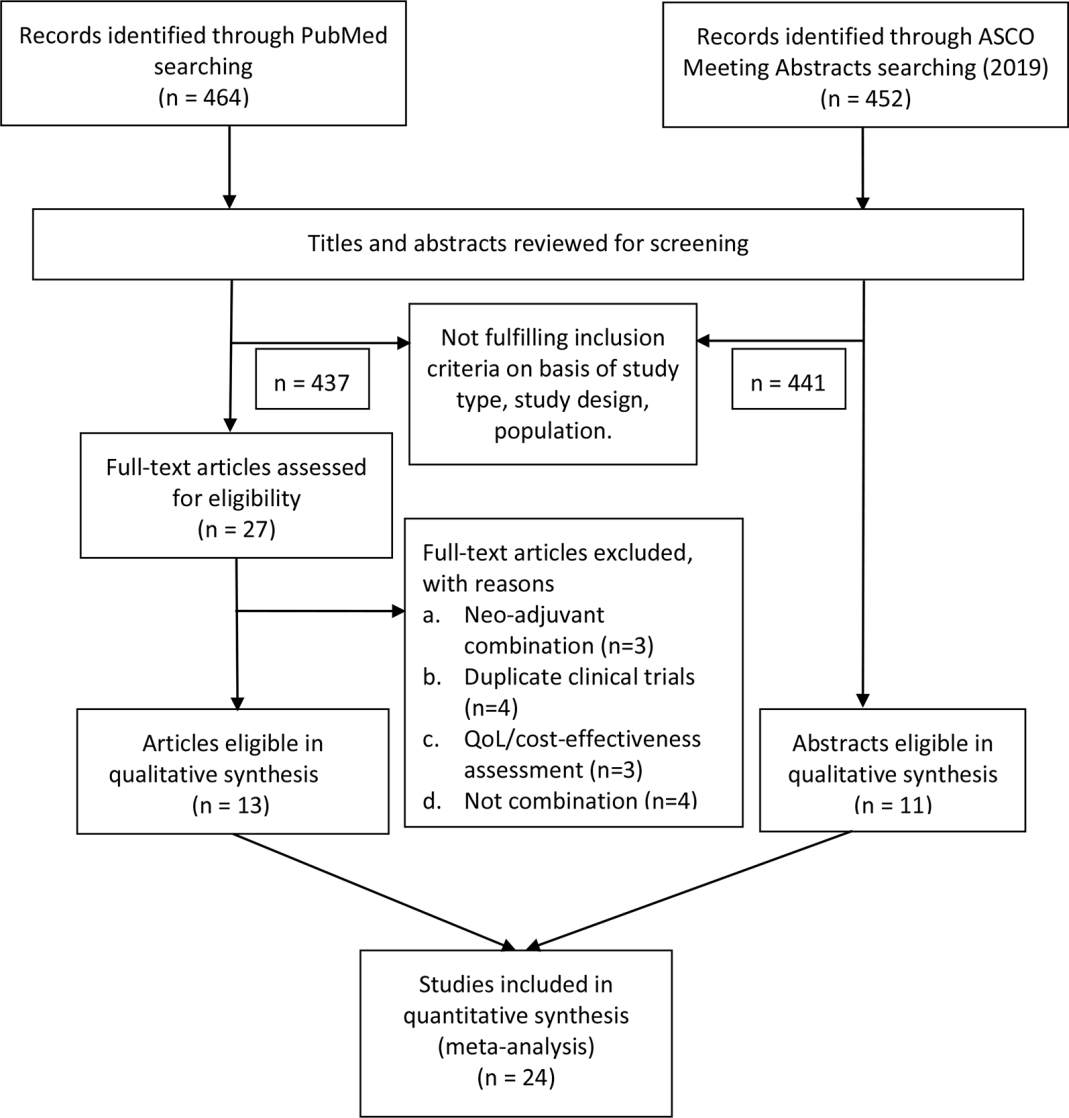
Flow diagram of the eligible trials included in this study. ASCO, American Society of Clinical Oncology; QoL, quality of life.

We applied the Population, Intervention, Comparator, Outcome, and Study design (PICOS) approach to identify eligible studies. Clinical trials (S) that investigated nivolumab and ipilimumab combination use (I, C) in advanced cancer patients (P), and provided information on ORR and high-grade AEs (O) were selected. We included all the prospective clinical trials that meet the following items: (1) investigating the combined use of nivolumab and ipilimumab in patients with advanced solid tumors; (2) with results that reported the ORR/the incidence of treatment-related grade 3–4 AEs/the number of treatment-related death; (3) the 2019 ASCO annual meeting abstracts were included if they meet the above two criteria. We excluded trials that: (1) involved combination regimens with other treatment modalities (e.g. nivolumab plus ipilimumab plus radiotherapy); (2) investigated the neo-adjuvant nivolumab combined with ipilimumab in cancer patients; (3) were quality of life analysis or cost-effective assessment of the trials; (4) the results didn’t report the specific number or rate of objective response and AEs data. Besides, case reports, editorials, letters and correspondences were excluded. Review and systematic review were screened for potential omitted qualified trials despite they were excluded from our study. In the event of duplicated trials, we selected the most recent trials into our study. Discrepancies regarding the inclusion and exclusion criteria were resolved by consensus (**Figure 1**).

### Data Extraction

The data were extracted by 1 reviewer (HX) primarily and were reviewed by another reviewer (PT) following the Preferred Reporting Items for Systematic Reviews and Meta-Analyses guidelines. Efficacy and safety data were separately extracted from trial results. The number of events (objective response, treatment-related grade 3 or more AEs) were recorded. Numbers of objective response were calculated as numbers of partial response + numbers of complete response. In addition, the frequency and spectrum of treatment-related grade 3–4 AEs and fatal AEs (i.e. one specific AE) were also recorded from publications (owing to the limited information on ASCO abstracts, they were not included in this analysis). Besides, information on first author name, ASCO abstracts number, study year, NCT number, phase, cancer type, doses and frequency of nivolumab plus ipilimumab combination, median follow-up duration were also recorded (**Table 1**). For those trials that had multiple arms, we only included the nivolumab plus ipilimumab combination arms and extracted data from each arm. Data were extracted by two reviewers independently and discrepancies were resolved by discussion.

**Table 1:** Baseline characteristics of trials included in this study.

Study	Year	NCT Number	Phase	Cancer Type	Combination Therapy Arms				Median Follow-up	Efficacy, TN	OR, N	Safety, N	Grade 3 or 4, N	FAEs, N
					NIVO		IPI							
					Dosage									
**Publications**														
Tawbi	2018	02320058	2	Melanoma	1 mg/kg	Q3W	3 mg/kg	Q3W	14	94	48	94	52	1
Omuro	2018	02017717	1	Glioblastoma	1 mg/kg	Q3W	3 mg/kg	Q3W	27.2	10	0	10	9	0
					3 mg/kg	Q3W	1 mg/kg	Q3W		20	2	20	6	0
Motzer^#^	2018	02231749	3	RCC	3 mg/kg	Q3W	1 mg/kg	Q3W	25.2	425	230	547	305	8
Long	2018	02374242	2	Melanoma	1 mg/kg	Q3W	3 mg/kg	Q3W	17	35	16	35	19	0
Hellmann	2018	02477826	3	Lung Cancer	3 mg/kg	Q2W	1 mg/kg	Q6W	11.2*	139	63	576	180	7
D’Angelo	2018	02500797	2	Sarcoma	3 mg/kg	Q3W	1 mg/kg	Q3W	14.2	38	6	42	6	0
Overman	2018	02060188	2	CRC	3 mg/kg	Q3W	1 mg/kg	Q3W	13.4	119	65	119	38	0
Wolchok^#^	2017	01844505	3	Melanoma	1 mg/kg	Q3W	3 mg/kg	Q3W	38	314	183	313	223	2
Hellmann	2017	01454102	1	Lung Cancer	3 mg/kg	Q3W	1 mg/kg	Q12W	12.8	38	18	38	14	0
					3 mg/kg	Q3W	1 mg/kg	Q6W	11.8	39	15	39	13	0
Hammers	2017	01472081	1	RCC	3 mg/kg	Q3W	1 mg/kg	Q3W	22.3	47	19	47	18	0
					1 mg/kg	Q3W	3 mg/kg	Q3W		47	19	47	29	0
					3 mg/kg	Q3W	3 mg/kg	Q3W		NA	NA	6	5	0
Hodi	2016	01927419	2	Melanoma	1 mg/kg	Q3W	3 mg/kg	Q3W	24.5	95	56	94	51	3
Antonia	2016	01928394	1/2	Lung cancer	1 mg/kg	Q3W	3 mg/kg	Q3W	12	61	14	61	18	2
					3 mg/kg	Q3W	1 mg/kg	Q3W	8.7	54	10	54	10	1
Wolchok	2013	01024231	1	Melanoma	0.3mg/kg	Q3W	3 mg/kg	Q3W	NA	14	3	14	6	0
					1 mg/kg	Q3W	3 mg/kg	Q3W		17	9	17	11	0
					3 mg/kg	Q3W	1 mg/kg	Q3W		15	6	16	7	0
					3 mg/kg	Q3W	3 mg/kg	Q3W		6	3	6	4	0
**ASCO**														
Abstr 2570	2019	02923934	2	Mixed	3 mg/kg	Q3W	1 mg/kg	Q3W	NA	53	17	60	19	0
Abstr 2613	2019	EudraCT 2016-003946-99	2	Mixed	3 mg/kg	Q2W	1 mg/kg	Q6W	4.3	20	1	NA	NA	NA
Abstr 4012	2019	01658878	1/2	HCC	1 mg/kg	Q3W	3 mg/kg	Q3W	24*	50	16	148	55	NA
					3 mg/kg	Q3W	1 mg/kg	Q3W		49	15			
					3 mg/kg	Q2W	1 mg/kg	Q6W		49	15			
Abstr 4517	2019	02982954	3b/4	RCC	3 mg/kg	Q3W	1 mg/kg	Q3W	6.47*	28	8	28	6	0
Abstr 4518	2019	03333616	2	Bladder Cancer	3 mg/kg	Q3W	1 mg/kg	Q3W	3.6	13	4	19	4	0
Abstr 6084	2019	03172624	2	Head and Neck Cancer	3 mg/kg	Q2W	1 mg/kg	Q6W	NA	32	2	32	4	0
Abstr 8563	2019	03083691	2	Lung Cancer	1 mg/kg	Q3W	3 mg/kg	Q3W	NA	18	7	20	NA	2
Abstr 9014	2019	02785952	3	Lung Cancer	3 mg/kg	Q2W	1 mg/kg	Q6W	17.4	123	22	125	48	5
Abstr 9522	2019	01585194	2	Melanoma	1 mg/kg	Q3W	3 mg/kg	Q3W	8.6	30	5	35	14	0
Abstr 11017	2019	02880020	2	GIST	240 mg	Q2W	1 mg/kg	Q6W	NA	12	1	12	4	0
Abstr 11064	2019	03219671	2	Sarcoma	240 mg	Q3W	1 mg/kg	Q6W	3.1	10	5	10	0	0

NIVO, nivolumab; IPI, ipilimumab; OR, Objective Response; TN, total number; FAEs, fatal adverse events; RCC, Renal Cell Carcinoma; HCC, Hepatic Cell Carcinoma; GIST, Gastrointestinal Stromal Tumor; ASCO, American Society of Clinical Oncology; Abstr, abstract; NA, not applicable.

^#^Adverse events data were collected from clinicaltrials.gov.

*Minimum follow-up.

### Statistical Analysis

For efficacy analysis, the number of patients available for efficacy assessment and the number of patients with objective response were recorded from each arm. For safety analysis, the number of patients available for safety assessment and the number of patients with grade 3–4 AEs or fatal AEs were also recorded from each arm. The observed ORR and incidence of treatment-related grade 3–4 or fatal AEs is reported by arm with 95% confidence intervals (CI). Fixed effects models or random effects models were selected according to the heterogeneity. Heterogeneity was assessed according to the *I* ^2^ value. The log-odds transformation and restricted maximum likelihood estimation were applied in all models. Besides, the 0.5 adjustment were applied to handle proportions equal to 0 or 1. Meta regression included four variables (sources [publications vs ASCO abstracts], sample size [≥ 100 vs <100], cancer type and different drug dose. Odds ratio [OR] and its corresponding 95% CI were calculated as exponentiate the results from the meta-regression models. Statistical significance was considered as two-side *P* <0.05. All analyses were conducted using the “meta-for” and “meta” package from R 3.6.0 (R project).

## Results

### Search Results and Study Characteristics

Four hundred sixty four studies and 452 abstracts were initially retrieved from PubMed search and from 2019 ASCO annual meeting abstracts, respectively. After applying our study selection criteria, 24 clinical trials including 13 trials from PubMed (Wolchok et al., 2013; Antonia et al., 2016b; Hodi et al., 2016; Hammers et al., 2017; Hellmann et al., 2017; Wolchok et al., 2017; D’Angelo et al., 2018; Hellmann et al., 2018; Long et al., 2018; Motzer et al., 2018; Omuro et al., 2018; Overman et al., 2018; Tawbi et al., 2018) and 11 trials from ASCO annual meeting (Bazhenova et al., 2019; Emamekhoo et al., 2019; Fischer et al., 2019; Klein et al., 2019; McGregor et al., 2019; Mielgo et al., 2019; Pelster et al., 2019; Singh et al., 2019; Tchekmedyian et al., 2019; Yau et al., 2019; Zer et al., 2019) were finally included in this meta-analysis. The detailed study selection flow diagram can be seen in **Figure 1**.

Of all the trials included, 4, 2, 13, 4 and 1 studies were phase1, phase 1/2, phase 2, phase 3 and phase 3b/4 clinical trial, respectively. For each trial we only included cohorts with nivolumab plus ipilimumab arm, which resulted in 2,114 and 2,674 patients were eligible for efficacy and safety analysis, respectively. The most common cancer types were melanoma (six clinical trials, nine cohorts), lung cancer (five clinical trials, seven cohorts) and renal cell carcinoma (three clinical trials, five cohorts). The most commonly selected dose combination was nivolumab 3 mg/kg plus ipilimumab 1 mg/kg every 3 weeks (N3I1-Q3W, 12 cohorts) and nivolumab 1 mg/kg plus ipilimumab 3 mg/kg every 3 weeks (N1I3-Q3W, 11 cohorts). The median follow-up duration ranged from 3.1 months to 27.2 months. The baseline characteristics of trials included in this study can be seen in **Table 1**.

### Objective Response Rate (ORR)

**Figure 2:**
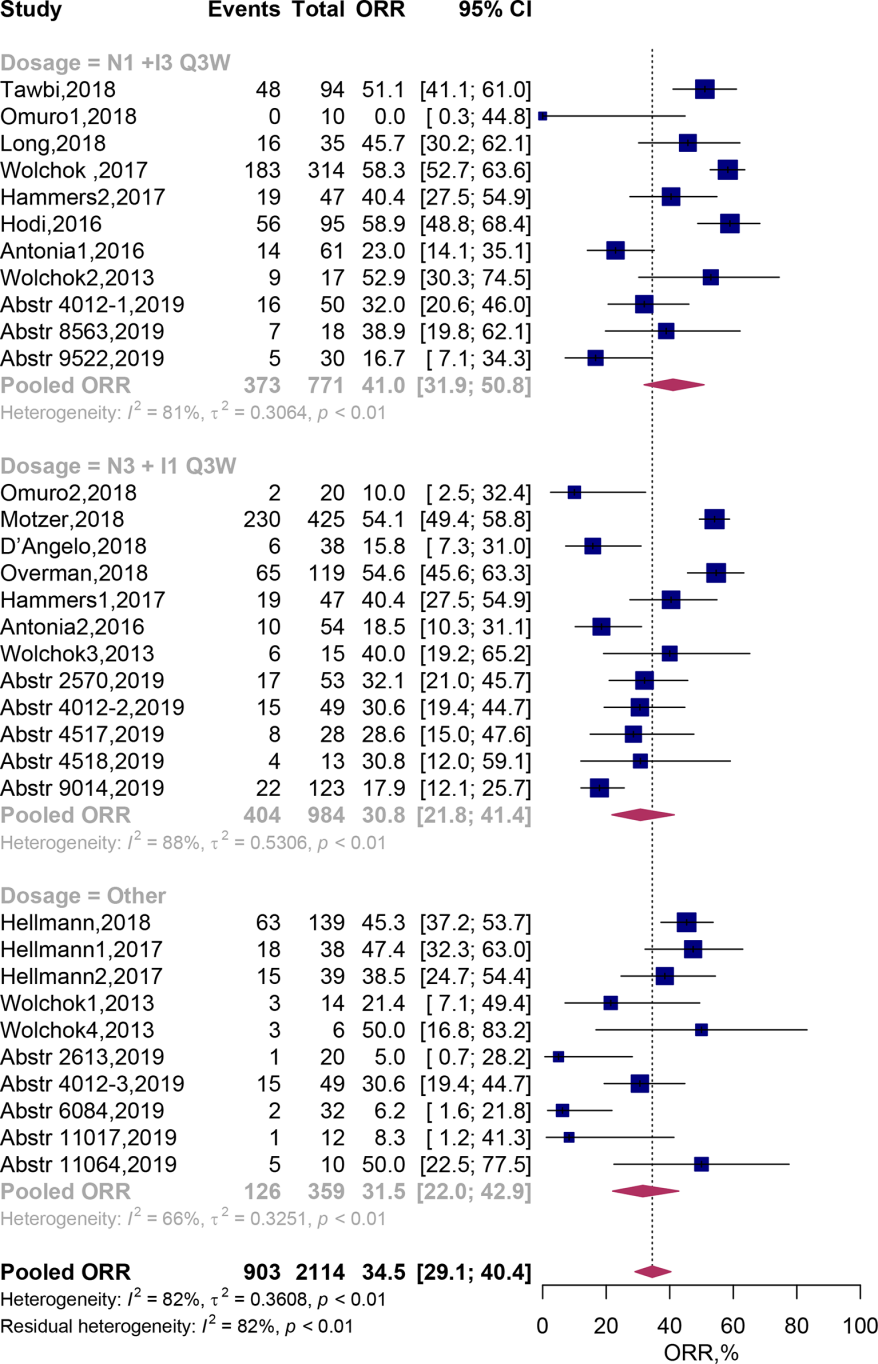
Forest plots of the objective response rate associated with nivolumab and ipilimumab combination treatment. ORR, Objective response rate; N, nivolumab; I, ipilimumab; CI, Confidence interval.

Twenty four clinical trials comprising 33 cohorts (2,114 patients) were available for the ORR analysis. By using random-effects models, the pooled analysis showed the ORR was estimated to be 34.5% (95% CI, 29.1–40.4%; **Figure 2**). Subgroup analysis showed that the predicted ORR was estimated to be 41.0% (95% CI, 31.9–50.8%) in N1I3-Q3W arms and 30.8% (95% CI, 21.8–41.4%) in N3I1-Q3W arms. Multivariate meta-regression analysis showed that there was no significant difference between these two drug doses (N3I1-Q3W vs N1I3-Q3W; OR, 0.72; 95% CI, 0.39–1.30; *P* = 0.275; **Table 2**). The test of residual heterogeneity (after excluding dose level moderator) among studies was statistically significant (Q = 170, *P* < 0.0001, *I* ^2^ = 81.59%). While no other study-level factors were found to be associated with ORR (**Supplementary Figure 1A**).

**Table 2:** Meta-regression model results for objective response rate and grade 3–4 adverse events.

Objective Response Rate			
Variable	Predicted Rate, % (95% CI)	Odds Ratio (95% CI)	*P*
**Dosage**			
N1 +I3 Q3W	41.0 (31.9–50.8)	Reference	
N3 + I1 Q3W	30.8 (21.8–41.4)	0.72 (0.39–1.30)	0.275
Other	31.5 (22.0–42.9)	0.92 (0.49–1.72)	0.786
**Cancer Type**			
Lung Cancer	31.4 (21.7–43.2)	Reference	
Melanoma	47.0 (38.2–56.0)	1.74 (0.90–3.35)	0.099
RCC	42.8 (31.6–54.8)	1.76 (0.86–3.62)	0.123
Other	24.8 (16.6–35.2)	1.21 (0.65–2.26)	0.541
**Grade 3–4 Adverse Events**			
**Variable**	**Predicted Incidence, (95% CI)**	**Odds Ratio (95% CI)**	***P***
**Dosage**			
N1 +I3 Q3W	55.9 (44.9–66.3)	Reference	
N3 + I1 Q3W	31.3 (22.7–41.4)	0.52 (0.32-0.87)	0.012
Other	34.1 (27.4–41.5)	0.64 (0.38-1.08)	0.098
**Cancer Type**			
Lung Cancer	31.9 (27.4–36.8)	Reference	
Melanoma	55.6 (46.4–64.5)	2.23 (1.32–3.75)	0.003
RCC	48.4 (34.4–62.6)	2.31 (1.40–3.80)	0.001
Other	28.4 (20.9–37.2)	1.10 (0.70–1.73)	0.666

RCC, Renal cell carcinoma; CI, Confidence interval.

In addition, when we categorized all trial arms according to cancer type, we found that the predicted ORR was achieved in 31.4% (95% CI, 21.7–43.2%) of lung cancer patients, 47.0% (95% CI, 38.2–56.0%) of melanoma patients, 42.8% (95% CI, 31.6–54.8%) of renal cell carcinoma and 24.8% (95% CI, 16.6–35.2%) of patients with other tumor types. Multivariate meta-regression analysis also didn’t reveal any significant difference between cancer types (all *P* > 0.05) (**Table 2**).

### Treatment-Related Grade 3–4 AEs

**Figure 3:**
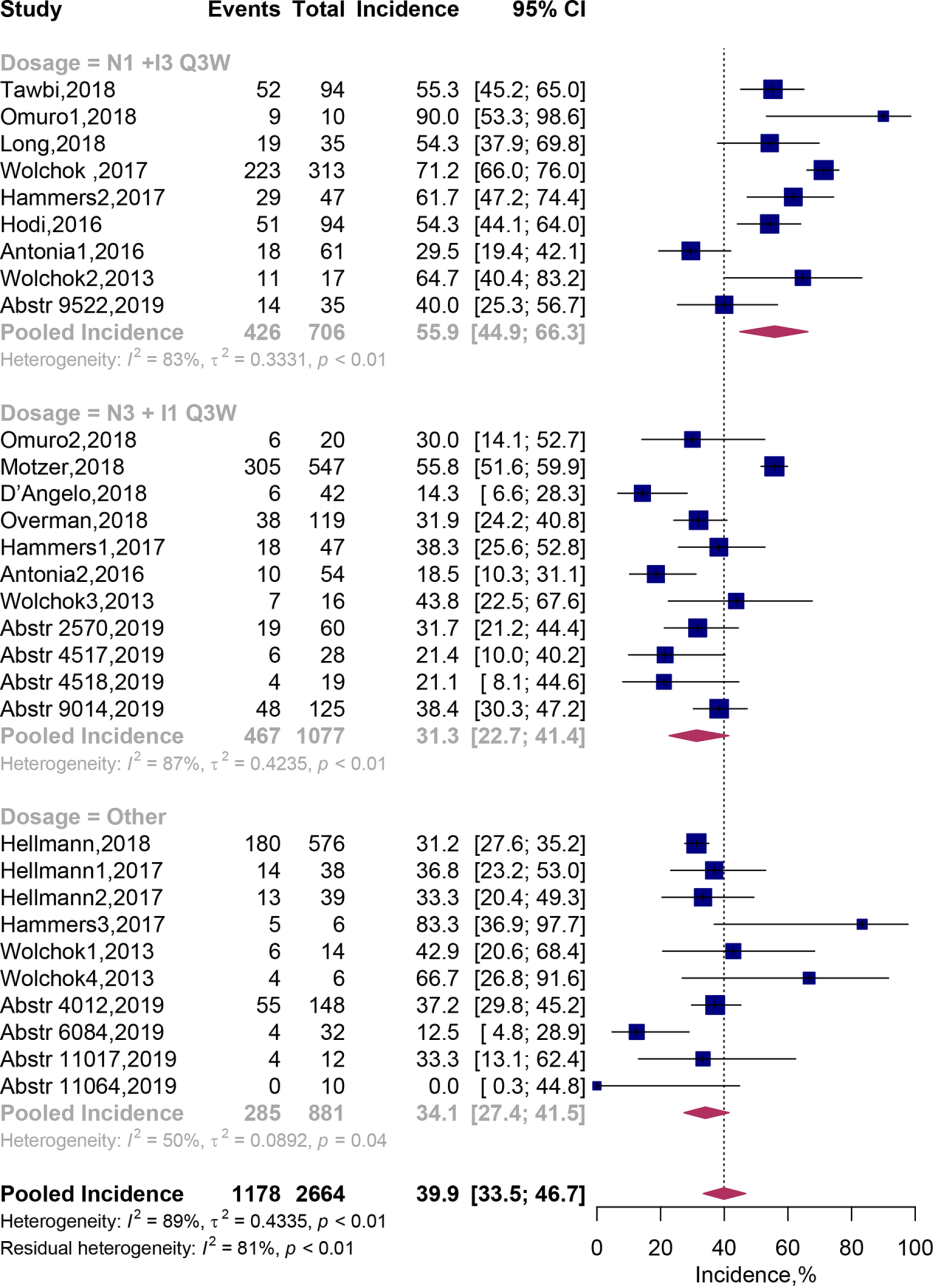
Forest plots of the incidence of grade 3–4 adverse events associated with nivolumab and ipilimumab combination treatment. CI, Confidence interval.

Thirty cohorts comprising 2,664 patients were available in assessment of treatment-related grade 3 or 4 AEs. By adopting random-effects models, pooled analysis suggested that grade 3–4 AEs related to the combination therapy occurred in 39.9% (95% CI, 33.5–46.7%) of patients (**Figure 3**). In addition, we recorded the spectrum of these high-grade AEs in our **Table 3**. It exhibited that the most commonly reported grade 3–4 treatment-related AEs were diarrhea [116 (5.28%)], colitis [87 (3.96%)], increased alanine aminotransferase [77 (3.51%)], Increased lipase [66 (3.01%)] and increased aspartate aminotransferase [65 (2.96%)].

**Table 3:** Incidence of specific grade 3–4 adverse events in included studies (not included ASCO meeting abstracts).

Grade3–4 AEs	Study													**Total Events (%)**
	Tawbi	Omuro	Motzer	Long	Hellmann	D’Angelo	Overman	Wolchok	Hellmann	Hammers	Hodi	Antonia	Wolchok	
**Skin**														
Pruritus	0	0	1	0	3	0	2	1	0	0	1	1;0	0	9 (0.41)
Rash	2	0	2	4	9	0	2	0	1;1	0	4	2;0	1;0;1;0	**29 (1.32)**
Maculopapular Rash	7	0	2	0	0	0	0	0	0;1	0	3	2;0	0	15 (0.68)
**Gastrointestinal**														
Colitis	7	2;1	10	7	3	0	0	31	1;2	0;7;0	12	1;1	1;1;0;0	**87 (3.96)**
Pancreatitis	1	0	3	0	0	0	0	2	1;0	0	2	0	0	9 (0.41)
Gastritis	1	0	1	0	2	0	0	1	0	0	0	0	0	5 (0.23)
Diarrhea	6	7;1	24	7	9	0	2	33	1;0	2;7;1	9	3;1	0;1;2;0	**116 (5.28)**
Vomiting	2	0	5	1	2	0	0	10	0;1	1;0;0	1	1;0	0;1;0;0	**25 (1.14)**
Nausea	2	3;0	8	1	3	0	1	9	0;1	1;0;0	1	1;0	0	**31 (1.41)**
Abdominal Pain	1	0	5	0	0	0	0	5	0	0	0	0	0	11 (0.50)
**Hepatic**														
Hepatitis	0	0	3	7	5	0	0	4	0	0	2	0	0	21 (0.96)
Acute Hepatitis	1	0	1	0	2	0	0	1	0	0	0	0	0	5 (0.23)
Autoimmune Hepatitis	1	0	0	0	0	0	0	6	0	0	0	0	0	7 (0.32)
**Endocrine**														
Adrenal Insufficiency	1	0	10	0	9	1	0	7	1;2	0	1	0;1	0	**33 (1.50)**
Hyperthyroidism	3	0	3	0	0	0	2	6	0	0	0	0	0	14 (0.64)
Hypothyroidism	1	0	2	0	2	0	1	2	0	0	0	1;0	0	9 (0.41)
Hypophysitis	5	0	14	1	1	0	0	8	0	0	2	0	0;0;0;1	**32 (1.41)**
Hypopituitarism	0	0	2	1	2	0	0	2	0	0	0	0	0	7 (0.32)
Adrenocortical Insufficiency Acute	0	0	2	0	0	0	0	1	0	0	0	0	0	3 (0.14)
Thyroiditis	0	0	3	0	0	0	0	2	0	0	1	0	0	6 (0.27)
**Respiratory**														
Pneumonitis	2	0	15	1	13	0	0	6	2;1	0	2	1;1	0	**46 (2.10)**
Dyspnoea	0	0	9	1	0	0	0	6	1;0	0	2	1;2	0	**22(1.00)**
Pulmonary Embolism	0	0;1	4	0	0	0	0	8	1;0	0	0	0	0	14 (0.64)
Respiratory Failure	0	0	2	0	0	0	0	3	0	0	0	0	0	5 (0.23)
Cough	0	0	2	0	0	0	0	1	0	0	0	0	0	3 (0.14)
**Musculoskeletal**														
Arthritis	0	0	1	0	0	0	0	1	0	0	0	0	0	2 (0.09)
Arthralgia	0	0	4	0	0	0	0	1	0	0	0	0	0	5 (0.23)
Myalgia	0	0	1	0	0	0	0	0	0	0	0	0	0	1 (0.05)
Back Pain	0	0	4	0	0	0	0	2	0	0	0	0	0	6 (0.27)
Pain in Extremity	0	0	1	0	0	0	0	1	0	0	0	0	0	2 (0.09)
Rhabdomyolysis	1	0	1	0	0	0	0	0	0	0	0	0	0	2 (0.09)
**Nervous system**														
Headache	3	0	3	0	0	0	0	2	0	0;1;2	2	0	0	13 (0.59)
Dizziness	0	0;1	0	0	0	0	0	0	0	0	1	0	0	2 (0.09)
Brain Edema	2	0	1	0	0	0	0	0	0	0	0	0	0	3 (0.14)
Syncope	1	0	2	0	0	0	0	1	0	0	1	0	0	5 (0.23)
Encephalitis	0	0	1	0	0	0	0	1	0	0	0	0	0	2 (0.09)
Meningitis	0	0	2	0	0	0	0	0	0	0	0	0	0	2 (0.09)
**Renal and Urinary Disorders**														
Acute Kidney Injury	1	0	8	0	0	0	0	7	1;0	0	0	0	0	17 (0.77)
Hematuria	0	0	2	0	0	0	0	0	0	0	0	0	0	2 (0.09)
Urinary Tract Infection	0	0	6	0	0	0	0	2	0	0	0	0	0	8 (0.36)
Renal Failure	0	0	0	0	0	0	0	3	0	0	0	0	1;1;1;0	6 (0.27)
Nephritis	0	0	0	1	0	0	0	1	0	0	0	0	0	2 (0.09)
Blood Creatinine Increased	0	0	5	0	0	0	0	0	0	1;1;0	1	0	0	8 (0.36)
**Cardiac**														
Myocarditis	0	0	1	0	0	0	0	0	0	0	0	0	0	1 (0.05)
Atrial Fibrillation	0	0	2	0	0	0	0	4	0	0	1	0	0	7 (0.32)
**Eye**														
Diplopia	0	0	2	0	0	0	0	1	0	0	0	0	0	3 (0.14)
Uveitis	1	0	0	0	0	0	0	1	0	0	0	0	0;2;0;0	4 (0.18)
**Vascular**														
Hypertension	0	0	0	0	0	0	0	1	0	0	0	0	0	1 (0.05)
Hypotension	2	1;0	4	0	0	0	0	2	0	0	2	0	0	11 (0.50)
**Hematologic**														
Anemia	1	0	6	0	9	1	0	3	0;1	0	0	0;1	0	**22 (1.00)**
Thrombocytopenia	0	0	0	0	0	0	0	1	0	0	0	0;1	0	2 (0.09)
**Meabolic**														
Hyperglycemia	0	0	0	0	0	0	0	5	0	0	2	1;0	0	8 (0.36)
Diabetes Mellitus	1	0	2	0	0	0	0	1	0;1	0	0	0	0	3 (0.14)
**Psychiatric**	0	0	0	0	0	0	0	0	0	0	0	0	0	
Confusional State	0	1;0	6	0	0	0	0	3	0	0	0	0	0	10 (0.46)
**General**														
Decreased Appetite	1	1;0	2	0	3	0	0	2	0	0	0	0	0	9 (0.41)
Fatigue	4	1;3	4	1	8	1	2	5	1;1	0;3;0	5	0	0	**39 (1.78)**
Pyrexia	0	0	18	0	0	0	0	26	0	2;0;1	3	0	0	**50 (2.28)**
Dehydration	2	0	7	0	0	0	0	8	0;1	0;2;0	2	0	0	**22 (1.00)**
**Investigations**														
Elevated ALT	15	2;2	9	2	4	2	8	3	0;1	2;10;0	10	0;1	2;3;0;1	**77 (3.51)**
Elevated AST	14	1;2	4	2	6	1	9	2	0;1	2;6;0	7	0;1	3;2;1;1	**65 (2.96)**
Increased Lipase Level	8	5;0	1	2	0	2	0	2	3;0	7;13;2	9	5;0	2;1;1;3	**66 (3.01)**
Increased Amylase Level	6	1;0	0	1	1	0	0	0	0	2;3;2	2	1;0	0;2;0;1	**22 (1.00)**
Increased Transaminases	2	0	3	0	0	0	0	8	0;1	0;2;0	1	0;1	0	18 (0.56)
GGT Increased	0	0	0	1	0	0	0	0	0	0	0	0;1	1;0;0;0	3 (0.14)
Hyponatremia	1	0	9	0	0	2	0	2	1;0	0	1	1;0	0	17 (0.77)
Hypokalemia	0	0	0	0	0	0	0	2	1;0	0	0	0	0	3 (0.14)

AEs, adverse events; ALT, alanine aminotransferase; AST, aspartate aminotransferase; GGT, γ-glutamyl transpeptidase.

“;” indicates multi-arms in one study by row; Bold values indicate the incidence of a specific adverse event exceeds 1%.

Subgroup analysis showed that the predicted incidence of treatment-related AEs was 55.9% (95% CI, 44.9–66.3%) in N1I3-Q3W arm and 31.3% (95% CI, 22.7–41.4%) in N3I1-Q3W arm. Multivariate meta-regression analysis showed that patients on the N3I1-Q3W arm were significantly less experience grade 3–4 AEs than patients on the N1I3-Q3W arm (OR 0.52; 95% CI, 0.32–0.87; *P* = 0.012). The test of residual heterogeneity among treatment arms was statistically significant (Q = 144, *P* < 0.0001, *I* ^2^ = 77.42%). Still, no other study-level factors were found to be associated with treatment-related grade 3–4 AEs (**Supplementary Figure 1B**).

In addition, when grouping cohorts by cancer type, the predicted incidence of grade 3–4 treatment-related AEs were 31.9% (95% CI, 27.4–36.8%) in lung cancer, 55.6% (46.4–64.5%) in melanoma and 48.4% (95% CI, 34.4–62.6%) in renal cell carcinoma. Incidence of treatment-related grade 3–4 AEs were significant higher in patients with melanoma (OR 2.23; 95% CI, 1.32–3.75; *P* = 0.003) and renal cell carcinoma (OR 2.31; 95% CI, 1.40–3.80; *P* = 0.001), when compared with lung cancer (**Table 2**).

### Fatal AEs

**Figure 4:**
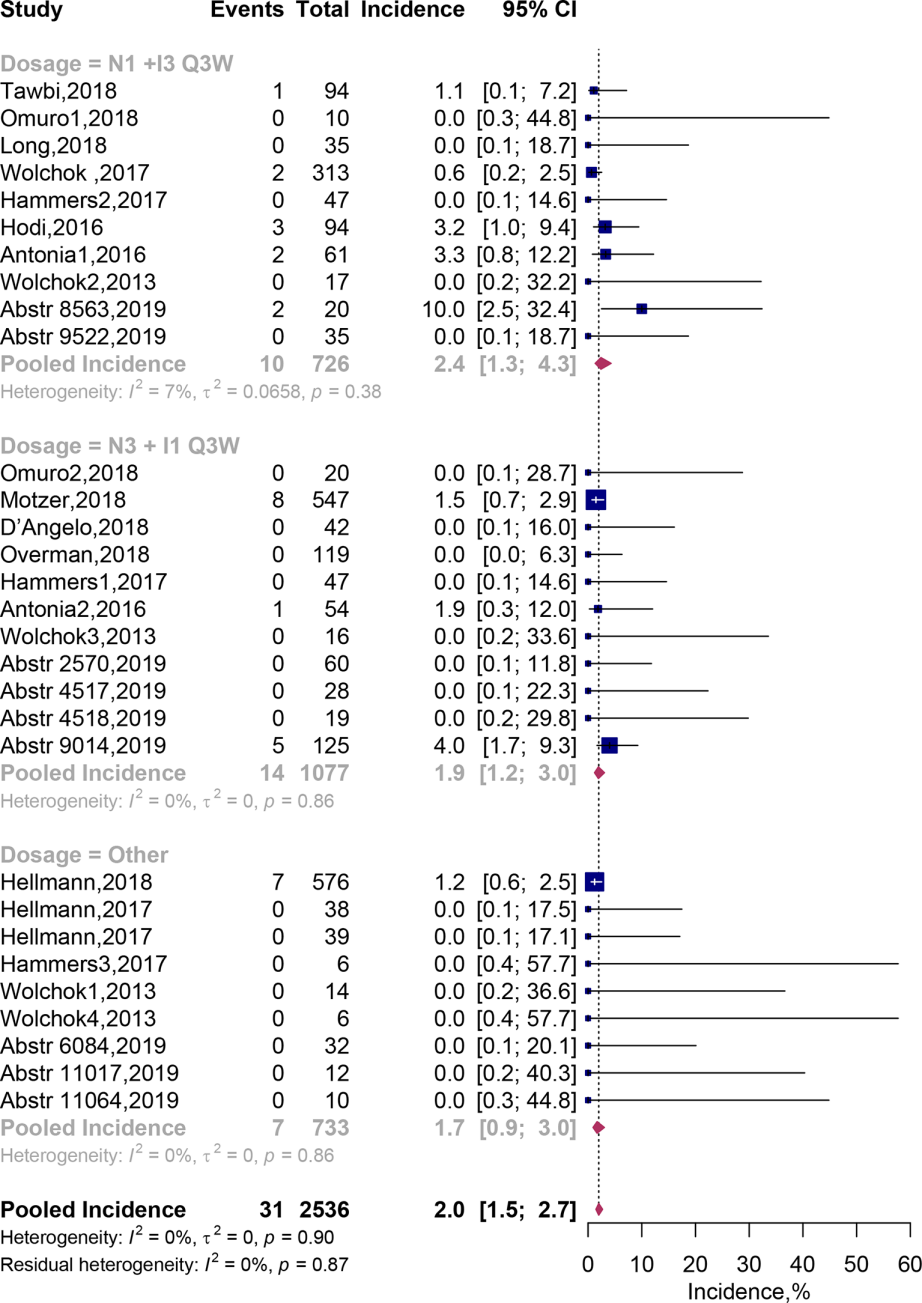
Incidence of fatal adverse events associated with nivolumab and ipilimumab combination treatment. Abstr, Abstract; CI, Confidence interval.

Of the 30 combination arms including 2,536 patients, fatal AEs were reported in 31 patients. Pooled meta-analysis using fixed-effects models showed that the incidence of fatal AEs was estimated to be 2.0% (95% CI, 1.5–2.7%; **Figure 4**). Incidence of treatment-related fatal AEs occurred about 2.4% (95% CI, 1.3–4.3%) on the N1I3-Q3W arm and 1.9% (95% CI, 1.2–3.0%) on the N3I1-Q3W arm. In addition, we listed each fatal AE in our **Table 4**. The results showed that incidence of fatal AEs was rare, mostly resulted from respiratory disorders [eight events (0.36%)] and cardiac disorders [seven events (0.32%)]. The most commonly reported fatal AEs was pneumonitis [six events [0.28%)]. The test of residual heterogeneity among treatment arms was not statistically significant (Q = 19, *P* = 0.8673) (**Supplementary Figure 1**).

**Table 4:** Incidence of specific fatal adverse events (grade 5) in included studies (not included ASCO meeting abstracts).

Fatal adverse events	Study												**Total Events (%)**
	Tawbi	Omuro	Motzer	Long	Hellmann	D’Angelo	Wolchok	Hellmann	Hammers	Hodi	Antonia	Wolchok	
**Cardiac disorders**													7 (0.32)
Myocarditis	1				1								2
Autoimmune Myocarditis							1						1
Ventricular Arrhythmia										1			1
Cardiac Insufficiency							1						1
Cardiac Tamponade					1								1
Circulatory Collapse					1								1
**Respiratory**													8 (0.36)
Pneumonitis			1		3					1	0;1		6
Immune-mediated Bronchitis			1										1
Lung Infection			1										1
**Hepatic**													2 (0.09)
Liver Toxic Effect			1										1
Liver Necrosis							1						1
**Renal**													2 (0.09)
Acute Tubular Necrosis					1								1
Renal Failure											1;0		1
**Endocrine**													2 (0.09)
Panhypopituitarism										1			1
**Gastrointestinal**													2 (0.09)
Lower Gastrointestinal Hemorrhage			1										1
**Hematologic**													2 (0.09)
Aplastic Anemia			1										1
The Hemophagocytic Syndrome			1										1
**Other**													2 (0.09)
Sudden Death			1										1
Myasthenia Gravis											1;0		1

This table only demonstrated the fatal adverse events reported by the included studies.

## Discussion

This meta-analysis investigated the efficacy and safety related to nivolumab and ipilimumab combination therapy in advanced cancer patients. The results showed that roughly 1/3 patients received combined nivolumab and ipilimumab therapy would achieve ORR; meanwhile, nearly 40% of the patients would occur grade 3–4 treatment-related AEs; treatment-related death was rare (2%). Moreover, we found that patients on the N3I1-Q3W arm had comparable ORR and significantly experience less grade 3–4 AEs than patients on the N1I3-Q3W arm, suggesting that the N3I1-Q3W regimen might be a better choice when we decided to administrate the combination therapies. By combining the latest clinical trial progress, we were able to draw the spectrum of severe and fatal treatment-related AEs associated with nivolumab plus ipilimumab regimen. Although several previous meta-analyses (Wang et al., 2018; Zhang et al., 2018; Wei et al., 2019) focused on the efficacy and safety of combination ICIs, our study is the first to investigate the estimated ORR and incidence of high-grade treatment-related AEs following the administration of nivolumab plus ipilimumab in solid tumors; moreover, our study is the first that we know of to compare the efficacy and safety from different drug dose level in combined nivolumab and ipilimumab therapy.

ICIs, including anti-CTL A-4, anti-PD-1 and anti-PD-L1 antibodies, are undoubtedly the most important progress in cancer treatment over the past decade. The indications for these drugs are continuing expanding across many clinical advanced settings, transforming many of the previous standard treatment modalities and bringing new dawn to traditionally “incurable” patients. Clinical evidence has shown the fact that nivolumab combined with ipilimumab could bring more durable responses compared with either agent alone in melanoma or lung cancer patients (Larkin et al., 2015; Antonia et al., 2016b; Hodi et al., 2016; Wolchok et al., 2017). A meta-analysis also concluded that combination ICIs could bring more ORR, progression-free survival (PFS) and overall survival (OS) benefits compared to control arms (Wei et al., 2019). The reason why we select ORR as the main indicator for efficacy of nivolumab and ipilimumab combination rather than PFS or OS is that most of the included studies didn’t meet the OS end-point and the definition of PFS is not consistent across various tumor types. The best ORR can be achieved 59% in melanoma in the trial conducted by Hodi et al. (2016). Our study also shows that 47% of melanoma patients receiving nivolumab and ipilimumab combination therapy can achieve complete or partial response. Then comes renal cell carcinoma, in which ORR could be achieved in around 43% of patients. Lung cancer patients only had 31% objective response benefit. Our results might be helpful in patients’ selection when the combination ICIs being an option.

Despite combined ICIs therapy showed its efficacy compared to ICI monotherapy in malignancies, however, the treatment-related AEs or immune-related AEs increased accordingly. In a comprehensive network meta-analysis performed by Xu et al., they provided a safety ranking of ICIs in cancer treatment (Xu et al., 2018). Their results demonstrated the pooled incidence of all grade AEs in ICIs combination was 57.7%, while in nivolumab was 14.4% and in ipilimumab was 25.2%. From their study we can know that combined ICIs could increase the AEs incidence, despite this 57.7% associated with ICIs combination might be inappropriate because they only included two trials. Another limitation is that they failed to show treatment-related grade 3–4 AEs associated with ICI combinations. By pooling 30 cohorts comprising 2,664 patients we were able to provide the relatively reliable incidence of grade 3–4 AEs (roughly 40%) related to combination use of nivolumab and ipilimumab, in comparison of 46% in nivolumab and 51% in ipilimumab from Xu’s study (Xu et al., 2018). From this point, the combination ICIs therapy might be acceptable and it wouldn’t increase the incidence of high-grade AEs compared with monotherapy. In addition, we exhibited the toxicity spectrum of grade 3–5 AEs associated with ICI combination. In a study conducted by Zhao et al. (2018), they demonstrated the most common treatment-related serious AEs were pneumonitis (8.2%), interstitial lung disease (5.6%) and colitis (3.6%) related to nivolumab therapy. While our study demonstrated the most commonly reported grade 3–4 treatment-related AEs were diarrhea (5.28%), colitis (3.96%) and increased alanine aminotransferase (3.51%) in the combination therapy. As for the fatal AEs related to ICI therapy, one meta-analysis found its incidence was 1.23% associated with ICIs combination therapy (Wang et al., 2018), and in our study this index was 2.0%. Regarding the spectrum of the fatal AEs related to ICIs combination, both of us showed the cardiac disorders and pneumonitis were the major cause of treatment-related death, though they rarely happened (< 1%).

In view of the drug doses during ICIs use, previous pooled analyses showed that ipilimumab 10 mg/kg every three weeks had a higher risk of grade 3–4 AEs than 3 mg/kg every three weeks (OR, 3.08; 95% CI, 1.52–6.32) (Xu et al., 2018), and no significant differences were found regarding fatal irAEs across different doses of ipilimumab (3 mg/kg vs 10 mg/kg for ipilimumab monotherapy; 1 mg/kg vs 3 mg/kg for combination ipilimumab therapy) (Wang et al., 2018). These results demonstrated incidence of high-grade AEs ipilimumab might be dose-dependent (Weber et al., 2012; Feng et al., 2013; Eggermont et al., 2016). This might explain our results that incidence of high-grade AEs was significant higher in N1I3-Q3W arm than in N3I1-Q3W arm. N3I1-Q3W is an ideal dose combination which didn’t eliminate the efficacy of combination therapy but rather decrease the incidence of grade 3–4 AEs.

Limitations of this study should be stated as well. We performed this meta-analysis from the study level; thus, we were unable to analyze the patient level variables such as patients’ sex and previous drug consuming that might affect the outcomes of our results. In addition, a significant proportion of the trials were from ASCO annual meeting abstracts with relatively short follow-up, which might lead to underestimation of their rates and overestimation of drug safety (Saini et al., 2014). Thirdly, published studies only reported the treatment-related AEs with an incidence above ≥1% or ≥5%, and there were only two studies posting their results in clinicaltrials.gov (Wolchok et al., 2017; Motzer et al., 2018); even though we’ve collected the data from the supplementary materials, some treatment-related grade 3–4 AEs might also be omitted in this study. Fourthly, we only analyzed two typical ICIs combination (nivolumab and ipilimumab) in this study, yet the efficacy or safety profile of other ICI combination [e.g. tremelimumab plus durvalumab (Antonia et al., 2016a; Calabro et al., 2018) and pembrolizumab plus ipilimumab (Long et al., 2017)] still remain unknow.

## Conclusions

In this comprehensive meta-analysis of 23 clinical trials, we provided the efficacy and complete toxicity profile and spectrum of treatment-related grade 3–4 AEs of combining nivolumab and ipilimumab in advanced cancer patients. We found that patients treated with N3I1-Q3W regimen had comparable ORR and experienced significantly less grade 3–4 adverse events than those who treated with N1I3-Q3W regimen. Our finding is of great importance in assisting clinical trial design and clinical medication choice.

## Data Availability Statement

The raw data supporting the conclusions of this manuscript will be made available by the authors, without undue reservation, to any qualified researcher.

## Author Contributions

HX and LY conceived and designed the study. HX, PT, JA, SZ, XL, XZ, LY, and QW screened the articles. HX, PT, and JA collated the data. HX, PT, JA, XZ, and SZ interpreted the data. SZ helped in editing the language. All authors drafted and revised critically the manuscript for important intellectual content. All authors gave final approval of the version to be published and have contributed to the manuscript. LY is the guarantor.

## Funding

This work was supported by the National Natural Science Foundation of China (Grant No. 81702536, 81974099), Programs from Science and Technology Department of Sichuan Province (Grant No. 2018JY0089 and No. 2018HH0153), the Prostate Cancer Foundation Young Investigator Award 2013, Chengdu Technological Innovation Research and Development Project (Chengdu science and Technology Bureau, 2018-YFYF-00131-SN), Young Investigator Award of Sichuan University 2017 (Grant No. 2017SCU04A17), and a grant from 1.3.5 project for disciplines of excellence, West China Hospital, Sichuan University (ZYGD18011). The funders had no role in patient selection, data extraction, statistical analysis or interpretation, writing of this article, or the decision to publish.

## Conflict of Interest

The authors declare that the research was conducted in the absence of any commercial or financial relationships that could be construed as a potential conflict of interest.

## Supplementary Material

Supplementary Figure 1 Funnel Plots for studies included in the meta-analysis for objective response rate (ORR), grade 3–4 adverse events (AEs) and fatal adverse events (FAEs).
