# Supplementary figures and images for: Antitumor Activity and Treatment-Related Toxicity Associated With Nivolumab Plus Ipilimumab in Advanced Malignancies: A Systematic Review and Meta-Analysis
Source: Front Pharmacol. 2019 Nov 4;10:1300. doi: 10.3389/fphar.2019.01300 (PMC6844121; doi:10.3389/fphar.2019.01300)

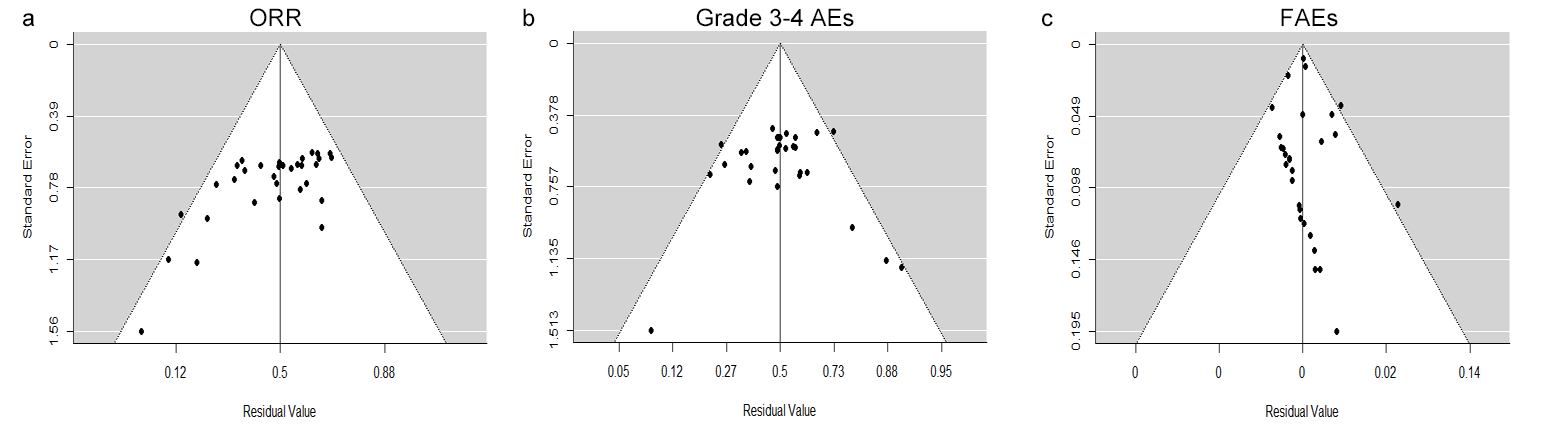

Supplement: Supplementary Figure 1 — Funnel Plots for studies included in the meta-analysis for objective response rate (ORR), grade 3–4 adverse events (AEs) and fatal adverse events (FAEs). [file Image_1.tif]
